# Spatial deformable transformer for 3D point cloud registration

**DOI:** 10.1038/s41598-024-56217-9

**Published:** 2024-03-06

**Authors:** Fengguang Xiong, Yu Kong, Shuaikang Xie, Liqun Kuang, Xie Han

**Affiliations:** 1 Shanxi Provincial Key Laboratory of Machine Vision and Virtual Reality, Taiyuan, 030051 China; 2 School of Computer Science and Technology, North University of China, Taiyuan, 030051 China; 3 Shanxi Province’s Vision Information Processing and Intelligent Robot Engineering Research Center, Taiyuan, 030051 China

**Keywords:** Computer science, Information technology

## Abstract

Deformable attention only focuses on a small group of key sample-points around the reference point and make itself be able to capture dynamically the local features of input feature map without considering the size of the feature map. Its introduction into point cloud registration will be quicker and easier to extract local geometric features from point cloud than attention. Therefore, we propose a point cloud registration method based on Spatial Deformable Transformer (SDT). SDT consists of a deformable self-attention module and a cross-attention module where the deformable self-attention module is used to enhance local geometric feature representation and the cross-attention module is employed to enhance feature discriminative capability of spatial correspondences. The experimental results show that compared to state-of-the-art registration methods, SDT has a better matching recall, inlier ratio, and registration recall on 3DMatch and 3DLoMatch scene, and has a better generalization ability and time efficiency on ModelNet40 and ModelLoNet40 scene.

## Introduction

Point cloud registration is a significant task in the field of computer vision and plays a crucial role in the fields of 3D reconstruction^1,2^, SLAM^3,4^ and autonomous driving^5,6^ and so on. The process of 3D point cloud registration techniques is to align multiple point clouds from different viewpoints or sensors into a same coordinate system. Due to the effects of noise, outliers, low overlap rate, etc., point cloud registration becomes a challenging problem. Therefore, it is of great theoretical and practical significance to implement a high-precision and robust point cloud registration algorithm.

The traditional Iterative Closest Point (ICP)^7^ is the most widely used rigid point cloud registration algorithm, which minimizes point-to-point or point-to-plane distances in the overlapping areas between point clouds, and alternately updates the corresponding relationship and transformation matrix between source point cloud and target point cloud. However, the main drawback of the ICP algorithm is that it easily converges to local optimums. To address this problem, J. Yang et al.^8^ proposed global optimal iterative nearest-point algorithm Go-ICP, which uses a branch-and-bound approach to search for the globally optimal registration at the cost of longer computing time.

With the continuous improvement of computer performance, deep learning-based methods have transformed traditional feature extraction methods. Choy et al.^9^ proposed FCGF, which used a ResUNet^10^ architecture built on 3D sparse convolution to extract features. However, FCGF is computationally expensive and implicitly decreases resolution^11^. Ao et al.^12^ proposed SpinNet to extract point cloud rotation invariance features. It consists of two modules, a spatial point transformer and a feature extractor, which make the network be able to learn local spatial features with strong robustness to finely register point cloud. X. Bai et al.^13^ proposed D3Feat including a KPConv^14^ feature extraction network which can be extended to deformable convolutions that learn to adapt kernel points to local geometry. Meanwhile, D3Feat proposed a novel keypoint selection method and a self-supervised detector loss to eliminate the impact of point cloud density on keypoints. On the basis of D3Feat, combined with the attention mechanism^15^, S.Huang et al.^16^ proposed PREDATOR which alternately uses self-attention and cross-attention mechanisms, and aggregates local and global information of point cloud. PREDATOR showing higher registration accuracy on 3DMatch scene dataset. Li et al.^17^ proposed Lepard which can register point cloud in deformable scenes. Lepard builds network using Transformer with self and cross attention, and ideas with differentiable matching. In rigid cases, Lepard combined with RANSAC^18^ and ICP demonstrates state-of-the-art registration recall. In deformable cases, Lepard also achieves higher non-rigid feature matching recall than the state-of-the-art method.

With the development of Transformer with self/cross attention in point cloud registration, the accuracy and precision of point cloud registration have a certain improvement. However, self/cross attention in Transformer is a global mechanism that focuses on all positions in the input features, which makes it difficult for the model to capture local features of the point cloud, and thereby reduces the robustness of registration to noise under low overlap scene. Meanwhile, self/cross attention calculates the weight of each position in the input features, which increases the computational complexity and affects registration efficiency. In recent years, some researchers proposed deformable attention^19,20^ on 2D image detection/recognition/classification to break through the limitation of self/cross attention. Deformable self/cross attention is a more flexible attention mechanism, whose core idea is to introduce deformability into the traditional self/cross attention mechanism, and allows the model to adaptively adjust the attention focus based on the specific situation of the input feature map. Deformable self/cross attention only focuses on a small group of key sampling points around the reference point, without considering the size of the feature map, and dynamically adjusts the weights of different positions in the input feature. In this way, it can obtain local features and improve the efficiency of feature extraction.

In this paper, we introduce deformable self/cross attention into point cloud registration and use spatial local positional relationships as the local position embeddings for deformable self-attention. Based on these, we propose Spatial Deformable Transformer (SDT) for point cloud registration. This approach enhances the ability to learn local geometric features through the SDT module, and reduces effectively the mismatching impact on the robustness of registration by constructing correspondence matrix based on Sinkhorn and Hungarian algorithm. Our main contributions are shown as follows.

- We propose a novel 3D point cloud registration network based on SDT to address point cloud registration under low overlap scenes.
- We construct a deformable self-attention module to interact local geometric spatial information within the point cloud to enhance the representation of features and make them easier to match.
- We construct a deformable cross-attention module to transfer features between point clouds to enhance feature discriminative capability of spatial correspondences.
- We design a balanced weighted loss function which uses focal loss between soft correspondence confidence matrix and the ground truth correspondence matrix as supervision to obtain more accurate hard matching correspondences between pairs of point clouds.

## Related Work

### Traditional point cloud registration

ICP^7^ is a classical traditional point cloud registration method, which finds the closest target points for each point in source point to generate 3D-3D correspondences and performs a least-squares optimization to compute rigid transformation between a pair of point clouds. The two steps are iteratively performed until a termination condition is satisfied. Many variants, such as Go-ICP^8^, Generalized-ICP^21^ and Sparse ICP^22^, have been proposed to increase its efficiency or o improve robustness to noise and mismatches. However, the main drawback of these methods is that they require proper initialization to converge to a good solution^23^. Another issue of ICP and its variants is poor robustness to outliers and partial overlaps that often occur in real-world data. Therefore, some traditional methods register point cloud by matching local shape descriptor and RANSAC algorithm. The representative shape descriptor includes PFH^24^, FPFH^25^, SHOT^26^, RoPs^27^, GASD^28^ etc. Nevertheless, the quality of such hand-craft descriptors can be affected by the point density and outliers^29^, and heavily rely on low-level geometrical attributes to compute orientations^30^.

### Learning-based point cloud registration

Recently, various deep learning^31–34^ approaches have been proposed for registration, such as PREDATOR^16^, REGTR^35^, PCRNet^36^, and so on. Learning-based Registration can be summarized into two categories: Feature learning-based methods and End-to-end learning-based methods. Unlike the traditional point cloud registration methods, Feature learning-based methods use deep neural network to learn a robust feature correspondence search, and then, the transformation matrix is ultimately determined through one-step estimation (e.g. RANSAC) without any iteration. PREDATOR employs an attention mechanism to extract contextual information for learning more distinctive feature descriptors and find soft-correspondences from overlap between a pair of point clouds. REGTR utilizes self-attention and cross-attention to directly predict a consistent set of final point correspondences. All these methods are using deep learning as a feature extraction tool and aim to estimate robust correspondences by the learned distinctive feature. The End-to-end learning-based methods solve registration problem with an end-to-end neural network. The input of the network is a pair of point clouds, and the output is the transformation matrix to align the pair of point clouds. The network not only can extract feature of point cloud, but also can estimate transformation. Different from the network of End-to-end learning-based method, the network of feature learning-based method is separate from the transformation estimation and focuses on feature learning. PCRNet uses PointNet to extract global features, and then connects these features together and provides them as input to the MLP network for regression transformation parameters. DeepGMR^37^ leverages a neural network to learn pose-invariant point-to-distribution parameter correspondences. Then, these correspondences are fed into the GMM optimization module to estimate the transformation matrix. DGR^38^ puts forward a 6-dimensional convolutional network architecture for internal likelihood prediction, and estimates the transformation through a weighted Procrustes module.

## Problem definition

Consider a pair of point clouds $P \in {\mathbb{R}}^{{N_{p} \times 3}}$ and $Q \in {\mathbb{R}}^{{N_{Q} \times 3}}$, we denote as source point cloud and target point cloud, respectively.$N_{P}$ and $N_{Q}$ denote the number of points in source point cloud ***P*** and target point cloud ***Q***, respectively. The objective of point cloud registration is to estimate an unknown rigid transformation consisting of a rotation ${\mathbf{R}} \in {\text{SO(3)}}$ and a translation ${\mathbf{t}} \in {\mathbb{R}}^{3}$, which aligns ***P*** to ***Q***.

## Methodology

**Figure 1:**
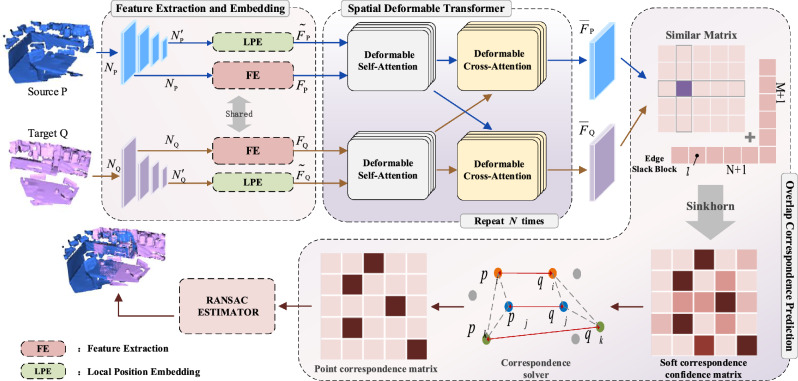
Main framework of our proposed point cloud registration. Feature extraction and embedding downsample the source point cloud *P* and the target point clouds *Q*, and learn features in multiple resolution levels and extract local position relationships from these point clouds as their local position embeddings, respectively. Spatial deformable transformer can enhance feature representation by deformable self-attention and can compare the similarity between two features by deformable cross-attention. Overlap correspondences prediction can estimate correspondences between these point clouds in the overlapping region by Sinkhorn and Hungarian algorithm.

Figure 1 illustrates our overall framework which consists of three main modules: feature extraction and embedding module, SDT module and overlapping correspondence prediction. In feature extraction and embedding module, it extracts feature of a pair of point cloud by a feature extraction network with shared weight, and we also compute local spatial relationships as local position embeddings between points of point cloud after downsampling. In SDT module, it first receives extracted feature and local position embeddings from feature extraction and embedding module, and then iteratively performs deformable self-attention and cross-attention whose purpose is to simulate the process of human browsing back and forth during matching. Deformable self-attention aims to make features more expressive for matching by enhancing local geometric feature representation of a point cloud, and deformable cross-attention aims to compare the similarity between a pair of point clouds by enhancing feature discriminative capability of spatial correspondences. In overlap correspondence prediction module, we first obtain a similarity matrix by matrix operations on the high- high-dimensional feature map from the previous module, and then we add edge slack block for the similar matrix and use Sinkhorn^39^ algorithm to obtain a soft correspondence confidence matrix, and we transform the soft feature correspondence into a one-to-one point correspondence through the utilization of the Hungarian algorithm^40^. Finally, RANSAC algorithm is employed to estimate the final transformation relationship between the source point cloud P and target point cloud Q.

### Feature extraction and local position embedding

#### Feature extraction

**Figure 2:**
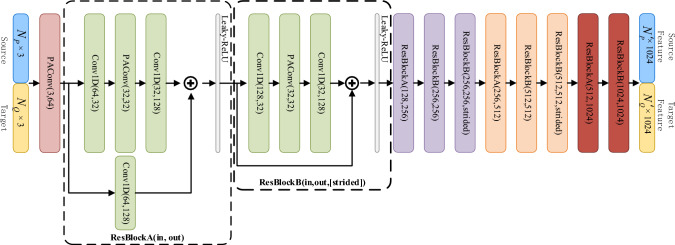
Feature extraction network structure. The dimension of original source point cloud is $\left( {N_{P} ,3} \right)$, and the dimension will turn to $\left( {N_{P} ,64} \right)$ after executing PAConv(3,64), and will turn to $\left( {N_{P} /4,128} \right)$ after executing a group of ResBlock marked with green color, and will turn to $\left( {N_{P} /16,256} \right)$ after executing a group of ResBlock marked purple color, and turn to $\left( {N_{P} /64,512} \right)$ after executing a group of ResBlock marked pink color, and turn to $\left( {N_{P} /64,512} \right)$ after executing a group of ResBlock marked red color which does not downsample the number of points since parameter strided in ResBlockB does not set to true.

To effectively utilize the input information of the original point cloud, the feature extraction adopts position adaptive convolution (PAConv)^41^ and residual network ResNet^42^ for multilevel resolution feature extraction and fusion. Unlike general convolutional networks, PAConv builds convolution kernels by dynamically assembling basic weight matrices stored in a weight bank, which can better handle irregular and disordered point cloud data and thus improve model registration performance. The backbone network architecture is illustrated in Fig. 2. Input point clouds can be expressed as ($N_{P}$, 3) and ($N_{Q}$, 3), where $N_{P}$ and $N_{Q}$ are the number of points in source point cloud ***P*** and target point cloud ***Q***, respectively, and 3 represents the coordinate dimension of each point. An original source/ target point cloud is input to the feature extraction network and passes through multi-layer ResBlockA and ResBlockB. In ResBlockA consists of a Conv1D convolution layer, a PAConv convolution layer, a Layer-Norm normalized layer, and a Leaky-ReLU activation layer and a shortcut Conv1D convolution layer. In ResBlockB, if parameter strided is set to true, PAConv will downsample the number of points to 1/4 of the number of points in upper-level structure, and the maxpool operation must be executed on the shortcut to ensure them be same dimension. We combine residual connections in the feature extraction backbone network and add multilevel resolved feature maps and convolutional results to achieve multilevel feature fusion. The correlation between ${\varvec{F}}_{P}$ and ${\varvec{F}}_{Q}$ of the point cloud ***P*** and ***Q*** is finally obtained. The dimension of feature map is ($N^{\prime}_{P}$, 1024) and ($N^{\prime}_{Q}$, 1024) respectively, where $N^{\prime}_{P}$ is 1/64 of the size of $N_{P}$ and $N^{\prime}_{Q}$ is 1/64 of the size of $N_{Q}$.

#### Local position embedding

**Figure 3:**
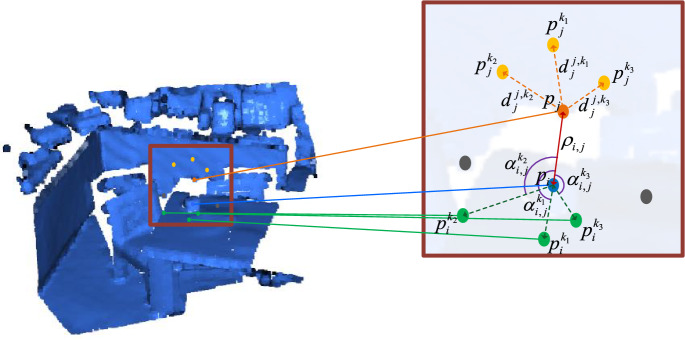
Local Position Relation.

The input of local position embedding (LPE) is the result by downsampling the source point cloud ***P*** and the target point cloud ***Q***. Based on^43^, the spatial position relation within the single point cloud is explicitly calculated and is taken as LPE of deformable self-attention in SDT. The spatial position relation of the point clouds is shown in Fig. 3.

In Fig. 3, $p_{i}$ and $p_{j}$ represent two points within a single point cloud, $p_{i}^{{k_{n} }}$ representing the *n-*th nearest neighbor of $p_{i}$ and $p_{j}^{{k_{n} }}$ representing the *n-*th nearest neighbor of $p_{j}$. We define a function ${\varvec{g}}_{{{\text{D}},i,j}}$ to describe the distance relationship between two points, and define $\rho_{i,j}$ to represent the Euclidean distance between $p_{i}$ and $p_{j}$, and $d_{i}^{{i,k_{t} }}$ to represent the distance between $p_{i}$ and its *n-*th.

nearest neighbor point, and $d_{j}^{{j,k_{t} }}$ to represent the distance between $p_{j}$ and its *n-*th nearest neighbor point, and $\tfrac{1}{n}\sum\limits_{t = 1}^{n} {d_{i}^{{i,k_{t} }} }$ to represent the average distance of $p_{i}$ and its *n* neighbor points (*n* is defined as 3 in this paper) , and $\tfrac{1}{n}\sum\limits_{t = 1}^{n} {d_{j}^{{j,k_{t} }} }$ to represent the average distance of $p_{j}$ and its *n* neighbor points, and $\sigma_{{\text{d}}}$ to represent a constant used to control the sensitivity of distance change, and $d_{t}$ to represent the dimension of the embedding vector. The function ${\varvec{g}}_{{{\text{D}},i,j}}$ is defined as follow

1 $$\left\{ {\begin{array}{*{20}l} g_{{{\text{D}},i,j,2k}} = \sin (\frac{{(\rho_{i,j} + \tfrac{1}{n}\sum\nolimits_{t = 1}^{n} {d_{j}^{{j,k_{t} }} } + \tfrac{1}{n}\sum\nolimits_{t = 1}^{n} {d_{i}^{{i,k_{t} }} } )/\sigma_{{\text{d}}} }}{{10000^{{2k/d_{{\text{t}}} }} }}) \\ g_{{{\text{D}},i,j,2k + 1}} = \cos (\frac{{(\rho_{i,j} + \tfrac{1}{n}\sum\nolimits_{t = 1}^{n} {d_{j}^{{j,k_{t} }} } + \tfrac{1}{n}\sum\nolimits_{t = 1}^{n} {d_{i}^{{i,k_{t} }} } )/\sigma_{{\text{d}}} }}{{10000^{{2k/d_{{\text{t}}} }} }}). \\ \end{array} } \right.$$

We define a function $${\varvec{g}}_{{{\text{A,}}i,j,ik}}$$ to describe the angle relation between three points, where $$\alpha_{i,j}^{{i_{t} }}$$ denotes the angle between vector $$\overset{\lower0.5em\hbox{$\smash{\scriptscriptstyle\rightharpoonup}$}}{{p_{i} p_{j} }}$$ and $$\overset{\lower0.5em\hbox{$\smash{\scriptscriptstyle\rightharpoonup}$}}{{p_{i} p_{i}^{{k_{t} }} }}$$, and $$\alpha_{i,j}^{{j_{t} }}$$ denotes the angle between vector $$\overset{\lower0.5em\hbox{$\smash{\scriptscriptstyle\rightharpoonup}$}}{{p_{i} p_{j} }}$$ and $$\overset{\lower0.5em\hbox{$\smash{\scriptscriptstyle\rightharpoonup}$}}{{p_{j} p_{j}^{{k_{t} }} }}$$, and $$\sigma_{{\text{a}}}$$ is a constant that controls the sensitivity to angle change, $$d_{t}$$ is the dimension of the embedding vector. The function $${\varvec{g}}_{{{\text{A,}}i,j,ik}}$$ is defined as follow

2 $$\left\{ {\begin{array}{*{20}l} g_{{{\text{A,}}i,j,ik,2x}} = \sin (\frac{{(\tfrac{1}{n}\sum\limits_{t = 1}^{n} {\alpha_{i,j}^{{i_{t} }} } + \tfrac{1}{n}\sum\limits_{t = 1}^{n} {\alpha_{i,j}^{{j_{t} }} )} /\sigma_{{\text{a}}} }}{{10000^{{2x/d_{{\text{t}}} }} }}) \\ g_{{{\text{A}},i,j,ik,2x + 1}} = \cos (\frac{{(\tfrac{1}{n}\sum\limits_{t = 1}^{n} {\alpha_{i,j}^{{i_{t} }} } + \tfrac{1}{n}\sum\limits_{t = 1}^{n} {\alpha_{i,j}^{{j_{t} }} )} /\sigma_{{\text{a}}} }}{{10000^{{2x/d_{{\text{t}}} }} }}). \\ \end{array} } \right.$$

Finally, the spatial position relation $\tilde{\user2{F}}$ between the $p_{i}$ and $p_{j}$ is defined as follow

3 $$\tilde{\user2{F}} = {\varvec{g}}_{{{\text{D}},i,j}} {\varvec{W}}_{{\text{D}}} + {\varvec{g}}_{{{\text{A}},i,j,ik}} {\varvec{W}}_{{\text{A}}} ,$$

where ${\varvec{g}}_{{{\text{D}},i,j}}$ is the distance relation between two points, ${\varvec{g}}_{{{\text{A,}}i,j,ik}}$ is the angle relation between three points, ${\varvec{W}}_{{\text{D}}}$ and ${\varvec{W}}_{{\text{A}}}$ are the projection matrices of the distance and angle relations, respectively, and the dimension of $\tilde{\user2{F}}$ is ($N^{\prime}$,$N^{\prime}$, 255).

### Spatial deformable transformer

SDT consists of a deformable self-attention module for enhancing local geometric feature expression capability and a deformable cross-attention module for transferring point cloud features whose aim is to compare the similarity between a pair of point clouds. It explicitly receives the local position embeddings and the high-dimensional features, and performs weighted aggregation of the features. In order to improve the computational efficiency, we change the dimension of feature map extracted from feature extract module from 1024 to 256d by linear projection. In the SDT module, these two deformable attention modules are executed iteratively for *n* times. We conduct extensive experiments and find that setting n to 4 can better and faster aggregate local features of point clouds. The outputs of SDT is $\overline{\user2{F}}_{P}$ and $\overline{\user2{F}}_{Q}$ according to (${\varvec{F}}_{P}$,$\tilde{\user2{F}}_{P}$) and (${\varvec{F}}_{Q}$,$\tilde{\user2{F}}_{Q}$) respectively, and their dimensions are ($N_{P}{\prime}$, 256) and ($N_{Q}{\prime}$,256) respectively.

#### (A) Deformable self-attention module

The original attention is used to describe the degree of autocorrelation of input information, and is represented by the attention weight matrix which is calculated by the query vector (*Query*, *Q*), key vector (*Key, K*), value vector (*Value, V*). Usually, V is weighted based on the relative importance of Q and K to obtain the attention matrix which can be expressed as follow

4 $$Attention({\varvec{Q}},{\varvec{K}},{\varvec{V}}) = softmax(\frac{{{\varvec{QK}}^{{\text{T}}} }}{{\sqrt {d_{{\text{k}}} } }})\user2{V,}$$

where $d_{{\text{k}}}$ is the dimension of the key vector. Attention is also called self-attention if *Q*, *K* and *V* comes from a same feature $\tilde{\user2{F}}$.

Different from self-attention, deformable self-attention^20,44^ predicts *k* position offsets according to query vector ***Q***, and calculates attention score according to ***Q*** and those *k* position of ***K*** and ***V***. In this paper, we use continuous position bias (CPB) method proposed in Swin Transformer V2^45^ to generate spatial deformation offset ${\varvec{B}}_{i,j}$ which improves the model's ability to capture local geometric information. ${\varvec{B}}_{i,j}$ is defined by the following formula

5 $${\varvec{B}}_{i,j} (\Delta x,\Delta y) = {\varvec{G}}_{i,j} (\Delta x,\Delta y),$$

where ${\varvec{G}}_{i,j}$ is by default a narrow network with one layer of ReLU activation function between two layers of MLP, and ${\varvec{B}}_{i,j}$ is the relative position offset between the query vector ***Q*** at $p_{i}$ and the key vector ***K*** at $p_{j}$*.*

**Figure 4:**
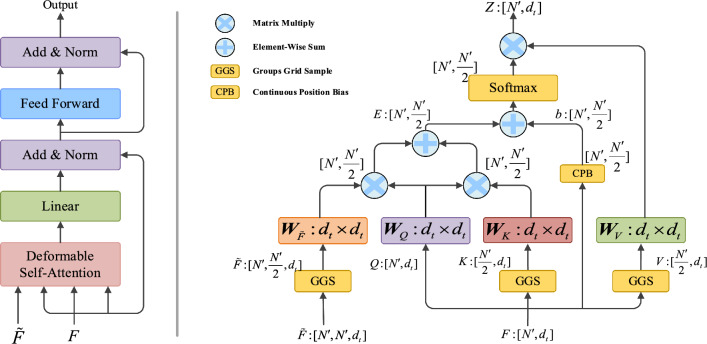
Deformable Self-Attention Module.

In the following, we describe the computation for (${\varvec{F}}_{P}$,$\tilde{\user2{F}}_{P}$) and the same goes for (${\varvec{F}}_{Q}$,$\tilde{\user2{F}}_{Q}$). Deformable self-attention performs a grouping strategy^46,47^ on the high-dimensional features ${\rm X} \in {\mathbb{R}}^{{\left| {F_{p} } \right| \times d_{t} }}$ to obtain ***Q***, ***K*** and ***V***, and perform groups grid sample^48^ on local position embeddings to obtain ***G***. By performing respectively dot product between ***Q*** and ***K***, ***Q*** and ***G***, and then adding it to the spatial deformation offset ${\varvec{B}}_{i,j}$, we obtain the attention score ${\varvec{e}}_{i,j}$ in Deformable Attention

6 $${\varvec{e}}_{i,j} = \frac{{({\varvec{x}}_{i} {\varvec{W}}_{Q} )({\varvec{x}}_{j} {\varvec{W}}_{K} + {\varvec{g}}_{i,j} {\varvec{W}}_{G} )^{{\text{T}}} }}{{\sqrt {d_{{\text{t}}} } }} + {\varvec{B}}_{i,j} ,$$

where ${\varvec{g}}_{i,j} \in {\mathbb{R}}^{{\left| {\rm X} \right| \times d_{t} }}$ denotes local position embedding between $p_{i}$ and $p_{j}$,${\varvec{W}}_{Q}$,${\varvec{W}}_{K}$,${\varvec{W}}_{G}$ are the projection matrix of the ***Q***, ***K*** and ***G*** respectively, and $d_{{\text{t}}}$ is the dimension of the input vector. Based on the obtained attention scores, the output feature matrix ${\varvec{z}}_{i}$ of deformable self-attention is the weighted sum of all projected input features

7 $${\varvec{z}}_{i} = \sum\limits_{j = 1}^{{|{\text{X}}|}} {{\varvec{a}}_{i,j} ({\varvec{x}}_{j} {\varvec{W}}_{V} )} ,$$

where ${\varvec{a}}_{i,j}$ denotes the weight coefficients computed by a row-wise softmax on the attention score ${\varvec{e}}_{i,j}$, and ${\varvec{W}}_{V}$ denotes the projection matrix of ***V***. Figure 4 shows deformable self-attention module, in which left part is the construction of deformable self-attention and right part is the computation graph of deformable self-attention.

The deformable self-attention module transforms the global feature interactions in traditional self-attention into local feature interactions which adapt to different geometric constructure of point cloud. By the deformable self-attention module, the model can adaptively learn the local geometric spatial information within the point cloud to enhance the representation of features and hence improve the accuracy of point-to-point matching.

#### (B) Deformable cross-attention module

**Figure 5:**
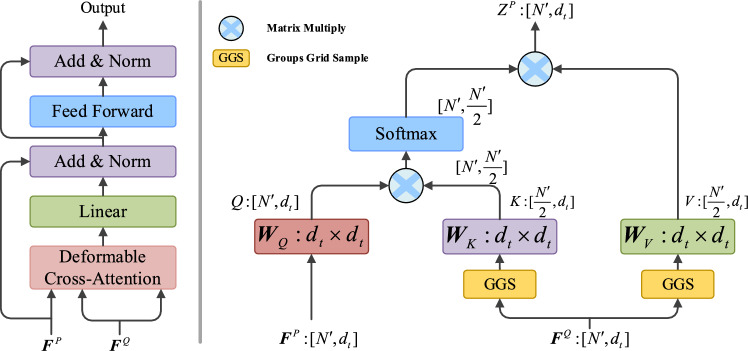
Deformable Cross-Attention Module.

A typical step in the point cloud registration task is deformable cross-attention module, which is used to exchange global features between points and then obtain the similarity between a pair of point clouds. Given the deformable self-attention feature matrix $f_{P}$ and $f_{Q}$ of the source point cloud ***P*** and target point cloud ***Q***, the feature correlation of $f_{P}$ relative to $f_{Q}$ can be expressed by ${\varvec{e}}_{i,j}$

8 $${\varvec{e}}_{i,j} = \frac{{({\varvec{f}}_{P,i} {\varvec{W}}_{Q} )({\varvec{f}}_{Q,j} {\varvec{W}}_{K} )^{{\text{T}}} }}{{\sqrt {d_{{\text{t}}} } }},$$

where ${\varvec{W}}_{Q}$,${\varvec{W}}_{K}$ are the projection matrices of the query vector ***Q*** and the key vector ***K*** respectively, and $d_{{\text{t}}}$ is the dimension of the input vector. Then, deformable cross-attention feature matrix ${\varvec{z}}_{P,i}$ of $f_{P}$ relative to $f_{Q}$ can be denoted as follow

9 $${\varvec{z}}_{P,i} = \sum\limits_{j = 1}^{|Q|} {{\varvec{a}}_{i,j} ({\varvec{f}}_{{{\text{Q, }}j}} {\varvec{W}}_{V} )} ,$$

in which ${\varvec{a}}_{i,j}$ is computed by a row-wise softmax on the attention score ${\varvec{e}}_{i,j}$, and ${\varvec{W}}_{V}$ denotes the projection matrix of ***V***. Figure 5 shows deformable self-attention module, in which left part is the construction of deformable cross-attention and right part is the computation graph of deformable cross-attention. The deformable cross-attention feature matrix of $f_{Q}$ relative to $f_{P}$ are computed in the same way, resulting in a more robust and discriminative feature representation after feature interactions.

### Overlap correspondence prediction

Overlap correspondence prediction module receives the output of SDT $\overline{F}_{{\text{P}}}$ and $\overline{F}_{{\text{Q}}}$, and unifies their dimensions as (max($N^{\prime}_{P}$,$N^{\prime}_{Q}$), 256) by bilinear interpolation^49^. So, cosine similarity matrix ***S*** can be defined as follow

10 $$S_{i,j} = \frac{{f_{i}^{P} \cdot f_{j}^{Q} }}{{\left| {f_{i}^{P} } \right|\left| {f_{j}^{Q} } \right|}},$$

where $f_{i}^{P}$ and $f_{j}^{Q}$ denote respectively a feature in $\overline{F}_{{\text{P}}}$ and $\overline{F}_{{_{{\text{Q}}} }}^{{\text{T}}}$. Before generating cosine similarity matrix ***S***, we can normalize respectively each feature in $\overline{F}_{{\text{P}}}$ and $\overline{F}_{{_{{\text{Q}}} }}^{{\text{T}}}$, so the value of $\left| {f_{i}^{P} } \right|$ and $\left| {f_{j}^{Q} } \right|$ are both 1, and ***S*** also can be defined as follow

11 $$S_{i,j} = f_{i}^{P} \cdot f_{j}^{Q} .$$

**Figure 6:**
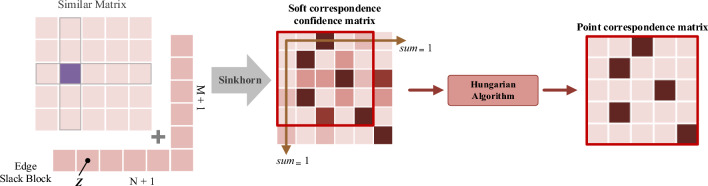
Process of overlap correspondence prediction.

We can obtain initially correspondences between source point cloud ***P*** and target point cloud ***Q*** from cosine similarity matrix ***S*** following a certain principle, for example $f_{i}^{P}$ and $f_{j}^{Q}$ are a pair of points if the value of $S_{i,j}$ is greater than a certain threshold. However, this approach will make a feature point in $\overline{F}_{{\text{P}}}$ correspond to multiple feature points in $\overline{F}_{{\text{Q}}}$, and will raise a lot of mismatching pairs which can decrease accuracy and robustness of registration. In response to the above issues, based on Dustbin mechanism of SuperGlue^50^, we add Edge Slack Block to normalized cosine similarity matrix ***S***, and utilize the Sinkhorn algorithm on ***S*** to compute a soft correspondence confident matrix***.*** Finally, we use Hungarian algorithm on the soft correspondence confident matrix to obtain a hard one-to-one correspondence confident matrix $M_{C}$. The process of overlap correspondence prediction is shown as Fig. 6.

### Loss

Inspired by UTOPIC^51^, we construct a supervised loss function via real correspondences based on the $\alpha$-balance cross-entropy loss^20^. The formula of the $\alpha$-equilibrium cross-entropy loss is defined as follow

12 $$L(M_{C} ) = - \alpha \log (M_{C} ),$$

where $M_{c}$ is a confidence matrix and denotes point-to-point correspondences, $\alpha$ is the balancing factor that resolves the imbalance of correspondences, $\alpha \in [0,1][0,1]$ is used on the correct correspondences, and $1 - \alpha$ is used on the incorrect correspondences. Based on the $\alpha$-balance cross-entropy loss, we add modulation factor $(1 - M_{C} )^{\upgamma }$ to obtain the correct corresponding loss $L_{\alpha }$

13 $$L_{\alpha } (M_{c} ) = - \alpha (1 - M_{c} )^{\upgamma } M_{gt} \log (M_{c} )$$

Similarly, the incorrect corresponding loss $\overline{L}_{\alpha }$ is defined as follow

14 $$\overline{L}_{\alpha } (M_{c} ) = - (1 - \alpha )M_{c}^{\gamma } (1 - M_{gt} )\log (1 - M_{c} ).$$

Finally, we obtain the total loss $L(M_{c} )$ of the model as follow

15 $$L(M_{c} ) = \sum\limits_{i = 1}^{{N_{P}{\prime} }} {\sum\limits_{j = 1}^{{M_{Q}{\prime} }} ( } L_{\alpha } (M_{c} ) + \overline{L}_{\alpha } (M_{c} )),$$

where $N_{P}{\prime}$ and $N_{Q}{\prime}$ represent the number of points after downsampling of source point cloud ***P*** and target point cloud ***Q***, respectively,$L_{\alpha } ({\varvec{p}}_{{\text{t}}} )$ indicating the correct corresponding loss function and $\overline{L}_{\alpha } ({\varvec{p}}_{{\text{t}}} )$ is the incorrect corresponding loss function. For registration data in 3DMatch and 3DLoMatch scenarios, we set $\alpha$ as 0.25 and set $\gamma$ as 2 according to^52^. For registration data in Modelnet40 scenarios, we set $\alpha$ as 0.45 and set $\gamma$ as 2.5.

**Figure 7:**
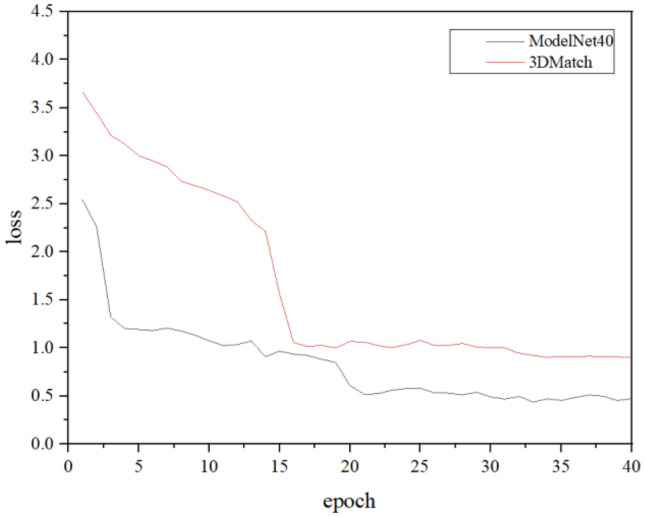
Curve of loss function.

Figure 7 shows curve of loss function for 40 epochs on Modelnet40 and 3DMatch under learning rate 0.0001 and decay 0.005. It is clearly note that the loss function continues to decrease for 3DMath and ModelNet40 as the number of epochs increases. Loss function on ModelNet40 converge to 0.5 after 20 epochs training and Loss function on 3DMatch converges to 0.9 after 16 epochs training.

## Experiments

### Experimental dataset and parameter setting

We evaluate SDT via the publicly available 3DMatch and ModelNet40 datasets. 3DMatch dataset contains 62 scenarios, in which 46 scenarios are used for training set, 8 scenarios are used for validation set, and 8 scenarios are used for testing. ModelNet40 dataset contains 40 CAD models from different classes, with the top 20 classes used for training and validating, rest 20 classes for testing. In our experiment, the data with overlap between 10 and 30% on 3DMatch are used as 3DLoMatch, and the average overlap is below 53.6% on ModelNet40 are used as ModelLoNet40, and these data are used to test the effect of our proposed method in the low overlap scenario. During training, the AdamW optimizer is used with the initial learning rate set to 0.0001 and learning rate decay is used to reduce learning rate to ensure better model convergence after 40 epochs. The model was trained and tested via PyTorch framework on a server equipped with an NVIDIA GeForce RTX 3090 GPU.

### Model evaluation metrics

We evaluate our method on 3DMatch and ModelNet40 datasets, and generalize directly the training model to low-overlap 3DLoMatch and ModelNet40 datasets, and compare the indicators of our proposed method with state-of-the-art registration methods. Three metrics from^12^ are used to evaluate the performance of our proposed method on 3DMatch dataset: (1) FMR (Features Matching Recall), the fraction of point cloud pairs whose inlier ratio exceeds a certain threshold; (2) IR (Inlier Ratio), the fraction of estimated correspondences whose residuals are below a certain threshold under the ground-truth transformation; (3) RR (Registration Recall), the fraction of point cloud pairs whose transformation error is smaller than a certain threshold. We evaluate the performance of our proposed method on ModelNet40 dataset by (1) RRE (Relative Rotation Error), the geodesic distance between estimated and ground-truth rotation matrices; (2) RRE (Relative Translation Error), the geodesic distance between estimated and ground-truth translation vectors; (3) CD (Chamfer Distance), a sum of positive distances between a pair of aligned point clouds.

### Comparison of the experiments

**Figure 8:**
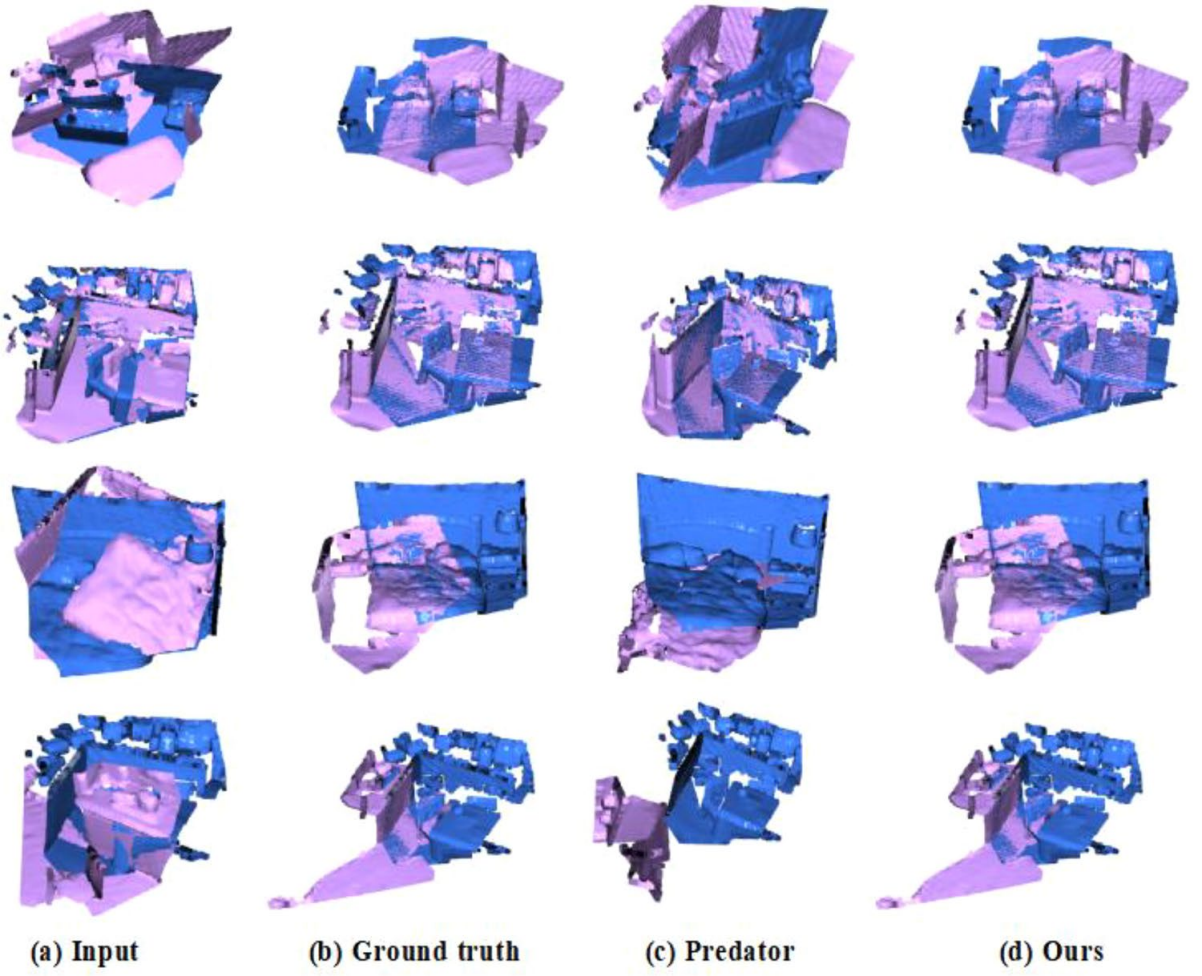
Registration Results on 3DMathc and 3DLoMatch.

Table 1 estimates the performance of SDT and state-of-the-art registration methods, such as FCGF, D3Feat Predator, CoFiNet ^53^. It is obvious from Table 1 that (1) On 3DMatch dataset, the FMR of our SDT is only slightly lower than CoFiNet, the IR of our SDT is lower than Predator, and the RR of our SDF outperforms all other methods; (2) On 3DLoMatch dataset, all metrics of our SDT outperform other methods, FMR of our SDT has a 3.7% higher than that of CoFiNet, IR of our SDT has a 2.2% higher than that of Predator, and RR of our SDT has a 3.9% higher than that of CoFiNet. All these experimental results show that our SDT can effectively register point clouds and is more robust and accurate to register point clouds with low-overlap. The registration graphs of our SDT and Predator on the 3DMatch and 3DLoMatch datasets are shown in Fig. 8, where diagrams in 1st and 2nd rows display some raw data from 3DMatch with 48.3% and 73.4% overlap and their registration results, and diagrams in 3rd and 4th rows display some raw data from 3DLoMatch with 29.0% and 21.5% and their registration results. It is obvious that our SDT can distinguish similar objects at different positions (see the comparison of Predator and SDT in the 3rd and 4th columns), and recognize small overlapping regions in complex environment thanks to local significant features obtained from the deformable self-attention and cross-attention.

**Table 1:** Comparison of FMR, IR and RR (%) of different methods.

Method	FMR		IR		RR	
	3DMatch	3DLoMatch	3DMatch	3DLoMatch	3DMatch	3DLoMatch
FCGF	97.4	75.9	56.9	22.0	87.3	41.7
D3Feat	95.8	67.4	40.7	15.5	84.9	46.9
Predator	96.6	77.9	**73.8**	37.8	90.6	62.4
CoFiNet	**98.1**	83.1	52.2	26.9	89.3	67.5
SDT (ours)	97.5	**86.8**	67.3	**40.0**	**91.0**	**71.4**

Significant values are in bold.

Due to structural differences for 8 test scenarios of 3DMatch and 3DLoMatch, the features obtained by different method are also extremely different for these different scenarios. Tables 2 and 3 show the comparison results of RR between different methods on 8 test scenarios of 3DMatch and 3DLoMatch, respectively. Experimental results show that our SDT outperforms other methods. In detail, on 3DMatch, RR of our SDT outperforms most scenarios, especially the hard scenarios such as Home_2 and Lab, and our SDT has a most mean RR; on 3DLoMatch, RR of our SDT is only lower that of CoFinet on Home_1 and Study, and has a most mean RR. These experimental results further show that our SDT together with overlap correspondences prediction is not only robust, but also accurate registration.

**Table 2:** Comparison of RR (%) of different methods on 3DMatch.

Method	3DMatch(RR)								
	Kitchen	Home_1	Home_2	Hotel_1	Hotel_2	Hotel_3	Study	Lab	Mean
FCGF	**98.0**	94.3	68.6	96.7	91.0	84.6	76.1	71.1	85.1
D3Feat	96.0	86.8	67.3	90.7	88.5	80.8	78.2	64.4	81.6
Predator	97.6	97.2	74.8	**98.9**	**96.2**	88.5	85.9	73.3	89.0
CoFiNet	96.2	**99.1**	73.2	95.8	91.2	84.6	89.9	84.4	89.3
SDT (ours)	97.6	96.8	**81.2**	98.4	89.1	**89.1**	**90.7**	**87.3**	**91.3**

Significant values are in bold.

**Table 3:** Comparison of RR (%) of different method on 3DLoMatch.

Method	3DLoMatch (RR)								
	Kitchen	Home_1	Home_2	Hotel_1	Hotel_2	Hotel_3	Study	Lab	Mean
FCGF	60.8	42.2	53.6	53.1	38.0	26.8	16.1	30.4	40.1
D3Feat	49.7	37.2	47.3	47.8	36.5	31.7	15.7	31.9	37.2
Predator	71.5	58.2	60.8	77.5	64.2	61.0	45.8	39.1	59.8
CoFiNet	74.1	**67.5**	64.4	81.7	65.5	63.1	**54.8**	68.1	67.4
SDT (ours)	**85.5**	64.0	**71.6**	**87.6**	**71.7**	**66.7**	54.0	**70.0**	**71.4**

Significant values are in bold.

In order to verify the robustness of our SDT at different sample points, the number of sampling points provided to network is gradually reduced in our experiment and RR of different methods are shown on Table 4. In all other cases, only when sample points on 3DModel are 1000, RR of Predator is slightly higher than our SDT, and whatever the number of sampling points on 3DLoMatch is, RR of our SDT outperforms all other methods. At the same time, the experimental results also show that our SDT is relatively robust to the number of different sampling points, even when the number of sample points is only 250.

**Table 4:** Comparison of RR (%) of different algorithms at different corresponding sampling points.

Method	3DMatch (RR)						3DLoMatch(RR)					
	5000	2500	1000	500	250	Mean	5000	2500	1000	500	250	Mean
FCGF	85.1	84.7	83.3	81.6	71.4	81.2	40.1	41.7	38.2	35.4	26.8	36.4
D3Feat	81.6	84.5	83.4	82.4	77.9	82.0	37.2	42.7	46.9	43.8	39.1	42.0
Predator	89.0	89.9	**90.6**	88.5	86.6	88.9	59.8	61.2	62.4	60.8	58.1	60.5
CoFiNet	89.3	88.8	88.7	87.8	87.0	88.3	67.3	66.9	64.5	63.1	62.0	64.8
SDT (ours)	**91.0**	**90.4**	90.3	**90.5**	**90.1**	**90.5**	**71.3**	**71.0**	**70.7**	**71.4**	**70.1**	**70.9**

Significant values are in bold.

**Figure 9:**
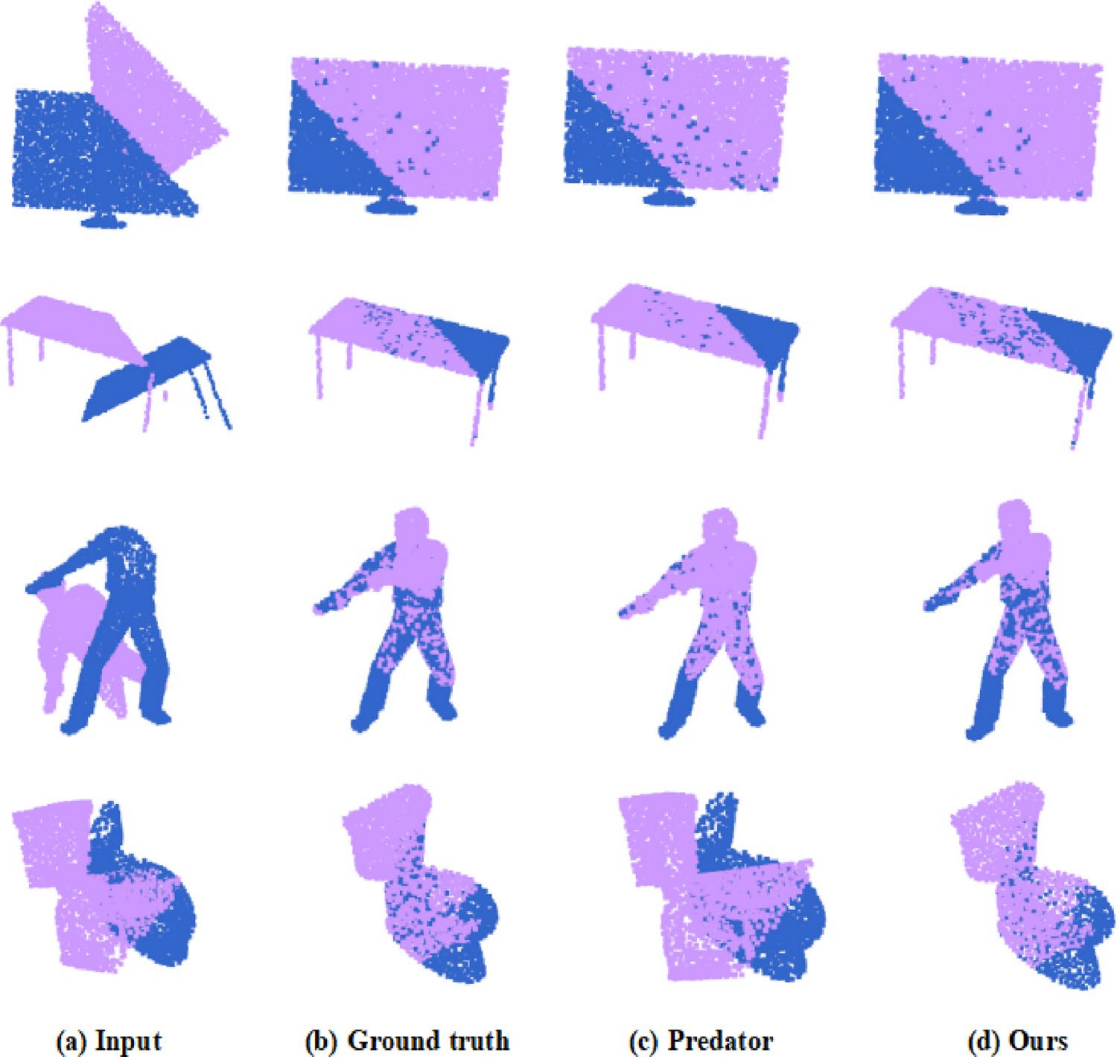
Registration Result on ModelNet40 and ModelLoNet40.

To further verify the generalization ability of our proposed method, we use first 20 categories of ModelNet40 dataset to train model and perform model test on left 20 unseen categories of ModelNet40 via trained model. Table 5 shows RRE, RTE, CD of our SDT and other methods on unseen categories. It is clearly shown that the performance of our SDT is as good as that of REGTR, and is better than that of DCP-v2^54^, RPM-Net^55^, and Predator. Experimental results also show that our SDT has strong generalization ability and better registration in low overlap scenarios. The registration graphs of our SDT and Predator on the ModelNet40 and ModelLoNet40 are shown in Fig. 9, where diagrams in 1st and 2nd rows display some raw data from ModelNet40 and their registration results, and diagrams in 3rd and 4th rows display some raw data from ModelLoNet40 and their registration results. It is obvious that our SDT outperform Predator (see the comparison of Predator and SDT in the 3rd and 4th row) on ModelLoNet40, which thanks to local significant features obtained from the deformable self-attention and cross-attention.

**Table 5:** Point cloud registration experiment with unknown object category.

Method	ModelNet40			ModelLoNet40		
	RRE	RTE	CD	RRE	RTE	CD
DCP-v2	11.975	0.171	0.0117	16.501	0.300	0.0268
RPM-Net	1.712	0.018	0.00085	7.342	0.124	0.0050
Predator	1.739	0.019	0.00089	5.235	0.132	0.0083
REGTR	**1.476**	0.014	**0.00079**	3.934	0.088	**0.0038**
SDT (ours)	1.614	**0.013**	0.00085	**3.915**	**0.078**	0.0041

Significant values are in bold.

### Ablations experiments

We perform ablation experiments on 3DMatch dataset to explore the impact of different components of our SDT on the registration results. Specifically, we test the effect of our network at three different modules.

1. DGCNN / no Sinkhorn. We use DGCNN to replace our FE in the process of feature extract and embedding, and Sinkhorn algorithm is removed from the overlap correspondences predict module.
2. DGCNN / Sinkhorn. We use DGCNN to replace our FE in the process of feature extract and embedding, and Sinkhorn algorithm is added to the overlap correspondences predict module.
3. Graph neural network / no Sinkhorn. In SDT module, graph neural network is used to replace SDT module, and Sinkhorn algorithm is removed from the overlap correspondences predict module.
4. Graph neural network / Sinkhorn. In SDT module, graph neural network is used to replace SDT module, and Sinkhorn algorithm is added to the overlap correspondences predict module.
5. Self/cross attention based/no-Sinkhorn. In SDT module, the original self-attention is used to replace the deformable self-attention in the SDT module, and the original cross-attention is used to replace the deformable cross-attention in the SDT module, and Sinkhorn algorithm is removed from the overlap correspondences predict module.
6. Self/cross attention-Based/ Sinkhorn. In SDT module, the original self-attention is used to replace the deformable self-attention in the SDT module, and the original cross-attention is used to replace the deformable cross-attention in the SDT module, and Sinkhorn algorithm is added to the overlap correspondences predict module.
7. Deform-self-Attention-Base/no-Sinkhorn. Sinkhorn algorithm is added to the overlap correspondences predict module.
8. Ours model. Deformable self-Attention is added to the SDT module, and Sinkhorn algorithm is added to the overlap correspondences predict module.

The results in Table 6 demonstrate that our SDT is essential for solving rigid transformations in low overlap scenarios. Among them, our proposed SDT has the best registration performance, whose FMR and RR has raised more 6% on 3DLoMatch. Meanwhile, the results in Table 6 also demonstrate that deformable attention and attention will be greater to improve FMR, IR and RR in point cloud registration comparing to graph neural network and DGCNN. Finally, it is worth noting that the addition of Sinkhorn algorithm in the DGCNN, graph neural network, self/cross attention and deformable self/cross attention can improve the registration effect.

**Table 6:** Comparison of FMR, IR and RR on network model.

Model	FMR		IR		RR	
	3DMatch	3DLoMatch	3DMatch	3DLoMatch	3DMatch	3DLoMatch
DGCNN/no Sinkhorn	93.2	80.7	47.5	25.2	82.1	64.2
DGCNN/Sinkhorn	94.0	81.6	54.7	30.3	84.3	67.1
Graph neural network/no Sinkhorn	95.1	82.1	48.2	26.6	84.3	64.7
Graph neural network/Sinkhorn	95.9	82.9	54.5	29.2	87.5	67.8
Self/Cross attention based/no Sinkhorn	96.1	83.3	48.8	30.5	86.7	66.9
Self/Cross attention based/Sinkhorn	97.0	83.8	64.3	35.7	88.7	68.2
Deformable self/cross attention Based/no Sinkhorn	97.2	85.9	65.1	37.1	90.4	70.1
SDT (ours)	**97.5**	**86.8**	**67.3**	**40.0**	**91.0**	**71.4**

Significant values are in bold.

Following that, we investigate the design of geometric structure embedding from the following aspects.

1. Number of neighbor points.We change the number of nearest neighbors to compute the triplet-wise distance/angular embedding of $p_{i}$ or $p_{j}$ in Fig. 3.
2. $\sigma_{{\text{d}}}$. It represent a constant used to control the sensitivity of distance change in formula (1) and we change the hyper-parameter to test its impact on registration performance.
3. $\sigma_{a}$. It represent a constant used to control the sensitivity of angular change in formula (2) and we change the hyper-parameter to test its impact on registration performance.

The results in Table 7 demonstrate that the impact of hyper-parameters on registration performance. According to Table 7, it is clear that the model with both distance and angular embeddings outperforms the model with simply distance embedding by a significant margin, which aligns with our goal. Increasing the number of neighbors will increases registration performance by providing exact structural information when the number of neighbor points is less than or equal to 3 , but registration performance whill decrease when the number of neighbor points is greater than 3, which shows the geometric structure formed by a reference point and its closest three neighbor points is the most robust to noise and the highest invariant to rigid transformation. Meanwhile, it is noted from Table 7 that the best results are obtained around 0.2 for $\sigma_{d}$ and 10°for $\sigma_{a}$ . A too small (where the embedding is too sensitive to distance changes) or too large (where the embedding neglects small distance variations) $\sigma_{d}$ could harm the performance, but the differences are not significant. And similar observations can be obtained for the angular changes $\sigma_{a}$. Nevertheless, all of these models outperforms pervious methods by a large margin, indicating that our proposed SDT is still robust to the distance/angular hyper-parameters.

**Table 7:** Comparison of FMR, IR and RR on hyper-parameters.

Model		FMR		IR		RR	
Name	Value	3DMatch	3DLoMatch	3DMatch	3DLoMatch	3DMatch	3DLoMatch
Number of neighbor points	0	92.0	82.1	63.1	35.3	84.7	66.1
	1	95.9	**84.1**	62.6	33.7	87.0	**68.5**
	**3**	**97.0**	83.8	**64.3**	**35.7**	**88.7**	68.2
	5	95.9	84.2	63.4	33.5	88.3	67.5
	7	96.1	83.8	60.0	33.8	85.8	66.5
$\sigma_{{\text{d}}}$	0.1	95.6	83.7	61.4	33.8	90.7	69.2
	0.2	96.4	83.8	63.0	37.4	**91.8**	69.5
	**0.3**	**97.5**	86.8	**67.3**	**40.0**	91.0	**71.4**
	0.4	96.1	**87.0**	65.3	36.0	87.8	70.0
	0.5	95.8	86.3	63.3	34.1	85.0	70.2
$\sigma_{{\text{a}}}$	5°	95.9	80.4	66.9	39.4	90.3	70.6
	**10°**	**97.5**	**86.8**	67.3	**40.0**	91.0	71.4
	15°	96.0	86.4	**68.7**	39.6	**91.8**	**73.2**
	20°	95.9	86.0	67.3	39.0	91.4	70.6
	25°	96.1	85.7	66.6	39.3	91.2	70.1

Significant values are in bold.

### Efficiency

We compare the inference time of several methods on a desktop computer equipped with an Intel I7-12700 CPU, an Nvidia GTX 3060 GPU, and 32G memory. Computational time is measured in seconds and calculated by averaging 100 results. As shown in Table 8, FCGF is the fastest method among these methods, and RPM-net, D3Feat are also faster than our proposed SDT, which is because their network is relatively simple and none of them adopt Transformer structure. Our proposed SDT is faster than DCP-v2, Predator, REGTR and CoFiNet, in which the former leverages deformable self/cross attention and the latter utilize Transformer structure with self/cross attention. From the results of the Table 8, it is clearly showed that Transformer with deformable self/cross attention (such as our proposed SDT) has a higher time efficiency than Transformer with self/cross attention.

**Table 8:** Inference time (in seconds).

Model	ModelNet	3DMatch
FCGF	**0.16**	**0.17**
RPM-Net	0.19	0.22
D3Feat	0.21	0.28
DCP-v2	0.74	0.85
Predator	0.30	0.38
REGTR	0.42	0.51
CoFiNet	0.83	0.94
SDT (ours)	0.24	0.29

Significant values are in bold.

## Conclusion

We propose a 3D point cloud registration method based on SDT. First, we propose a feature extraction and embedding module to extract basic features of point cloud and compute local spatial relationships between points in the point cloud as local positional embedding of basic feature, and formulate a SDT module to fuse and enhance above two kinds of information into new feature of point cloud by Self-Attention and Cross-Attention mechanisms. Second, we develop an overlap correspondence predict module to obtain correspondences between the pairwise point clouds by a series of handle for above new features of a pair of point clouds. Finally, we construct an $\alpha$-balance cross-entropy loss based on real correspondences of pairs of point clouds to train our unsupervised network, and we use outputs of this network to generate transformation matrices of pairs of point clouds via RANSAC algorithm. Extensive experimental results on the 3DMatch/3DLoMatch and ModelNet40/ModelLoNet40 demonstrate that our proposed method has high accuracy and strong robustness in solving point cloud registration problems in low overlap scenarios. Unfortunately, this leads to longer training and calculation times because the model uses the SDT module and the RANSAC approach simultaneously. Improving model performance, developing more efficient feature extraction and aggregation techniques, and extending our approach to more complicated scenarios need continue to study in the future.

### Ethical and informed consent

Data used in our study are publicly available, and ethical approval and informed consent were obtained in each original study.

## Data Availability

The datasets generated during and/or analyzed during our study are available from the corresponding author on reasonable request.
